# Deep Learning-Based Quantitative Assessment of Melamine and Cyanuric Acid in Pet Food Using Fourier Transform Infrared Spectroscopy

**DOI:** 10.3390/s23115020

**Published:** 2023-05-24

**Authors:** Rahul Joshi, Lakshmi Priya GG, Mohammad Akbar Faqeerzada, Tanima Bhattacharya, Moon Sung Kim, Insuck Baek, Byoung-Kwan Cho

**Affiliations:** 1 Department of Biosystems Machinery Engineering, College of Agricultural and Life Science, Chungnam National University, Daejeon 34134, Republic of Korea; 201860369@o.cnu.ac.kr (R.J.); akbar.faqeerzada@o.cnu.ac.kr (M.A.F.); btanima1987@gmail.com (T.B.); 2 Department of Multimedia, VIT School of Design (V-SIGN), Vellore Institute of Technology (VIT), Vellore 632014, India; lakshmipriya.gg@vit.ac.in; 3 Environmental Microbial and Food Safety Laboratory, Agricultural Research Service, United States Department of Agriculture, Powder Mill Road, BARC-East, Bldg 303, Beltsville, MD 20705, USA; moon.kim@usda.gov (M.S.K.); insuck.baek@usda.gov (I.B.); 4 Department of Smart Agricultural Systems, College of Agricultural and Life Science, Chungnam National University, Daejeon 34134, Republic of Korea

**Keywords:** melamine, cyanuric acid, multivariate analysis, one-dimensional convolutional neural network, Fourier transform infrared spectroscopy

## Abstract

Melamine and its derivative, cyanuric acid, are occasionally added to pet meals because of their nitrogen-rich qualities, leading to the development of several health-related issues. A nondestructive sensing technique that offers effective detection must be developed to address this problem. In conjunction with machine learning and deep learning technique, Fourier transform infrared (FT-IR) spectroscopy was employed in this investigation for the nondestructive quantitative measurement of eight different concentrations of melamine and cyanuric acid added to pet food. The effectiveness of the one-dimensional convolutional neural network (1D CNN) technique was compared with that of partial least squares regression (PLSR), principal component regression (PCR), and a net analyte signal (NAS)-based methodology, called hybrid linear analysis (HLA/GO). The 1D CNN model developed for the FT-IR spectra attained correlation coefficients of 0.995 and 0.994 and root mean square error of prediction values of 0.090% and 0.110% for the prediction datasets on the melamine- and cyanuric acid-contaminated pet food samples, respectively, which were superior to those of the PLSR and PCR models. Therefore, when FT-IR spectroscopy is employed in conjunction with a 1D CNN model, it serves as a potentially rapid and nondestructive method for identifying toxic chemicals added to pet food.

## 1. Introduction

Food fraud involving baby milk powder, meat, oils, and wine is prevalent in several areas of the food industry. Animal food has also been affected by contamination in the past in addition to products for human consumption, including mycotoxins in poultry feed and feed ingredients in Nigeria [1] and the mycotoxin contamination of feed and raw materials in China [2]. Melamine (1,3,5-triazine-2,4,6-triamine) is a widely used chemical responsible for the manufacturing of a variety of industrial products, such as melamine-formaldehyde resins, which are used to produce high-quality plastics, have flame-retardant properties, and can be used as a fertilizer. Cyanuric acid (2,4,6-trihydroxy-1,3,5-triazine), which is a melamine derivative and by-product, also has various industrial applications, such as household bleaches, disinfectants, industrial cleansers, and dishwasher compounds. In 2007, melamine was identified by the US Food and Drug Administration (FDA) when pet food imported from China killed and affected several pets after they consumed melamine-adulterated food [3]. Melamine was also found in baby milk powder samples imported from China in 2008, resulting in the illness of 294,000 individuals, including the hospitalization of 50,000 and the deaths of six children [4,5]. Because of the nitrogen-efficient qualities of both melamine and cyanuric acid, these chemicals are intentionally added by producers to increase the nitrogen content of food products [6,7]. Although the toxicity of melamine and cyanuric acid is very low when taken separately, they have a great affinity for one another when combined, leading to the creation of a barely soluble melamine–cyanurate complex [8,9]. Previous research demonstrated that this combination of melamine and cyanurate causes kidney and urinary bladder stones, severe chronic kidney inflammation, and renal failure via obstruction [10,11,12].

Typically, destructive chemical techniques, such as high-performance liquid chromatography (HPLC) [13], enzyme-linked immunosorbent assays (ELISAs) [14], zwitterionic hydrophilic interaction chromatography and tandem mass spectrometry [15], liquid chromatography-tandem mass spectrometry [16], and hydrophilic interaction liquid chromatography-ultraviolet detection (HILIC-UV) with an NH_2_ column [17], are used as conventional methods for detecting melamine or cyanuric acid in human foods and animal feed. Additionally, Montesano [18] used diode array detection technology (DAD) in conjunction with HPLC to examine the presence of melamine in protein supplements, with a high detection limit of 0.05–3.0 mg kg^−1^. These methods support the highest detection limits; however, the real-time monitoring of food and feed components is extremely challenging owing to their time-consuming, damaging, and complex sample preparation characteristics. Consequently, there is an urgent need for the development of rapid and nondestructive technologies to detect cyanuric acid and melamine in pet food.

Spectroscopy has developed into a promising and widely used approach in many disciplines, such as food quality, plant phytochemicals, and biological engineering, owing to advances in science and technology and the introduction of many sensing techniques. Previously, melamine and cyanuric acid have been identified using Raman and near infrared (NIR) spectroscopy. For example, Rodionova [19] used near infrared spectroscopy (NIRS) to predict the Mahalanobis distance (based only on spectral information) to detect samples containing melamine and cyanuric acid in samples of single feed ingredients (soybean meal, maize gluten, and wheat gluten) ranging from 0.5 to 6.0%, using adulterated synthesized samples. Furthermore, Nieuwoudt [20] utilized Raman spectroscopy to screen nitrogen-rich molecules, such as melamine, urea, ammonium sulfate, and sucrose in milk. However, the critical factors responsible for reducing the effectiveness of these two techniques are the generation of unwanted fluorescence effects in Raman spectroscopy and the generation of overtones and combination methods in NIR spectroscopy, which result in spectral broadness. Furthermore, the parameters to be predicted are not well-defined chemical entities, and developing calibrations to predict the ingredient composition in the final compound feed/food is a challenging task due to the heterogeneity of the matrices/formulas in which each ingredient is included. Nonlinear regression algorithms are advised for this purpose by Pérez Marín [21,22,23].

Fourier transform infrared (FT-IR) spectroscopy is a plausible solution for this flaw, because it allows for quick analysis, nondestructive measurement, easy sample preparation, and examination of the fundamental vibrations of molecules. Several studies have been conducted that clearly represent the potential of FT-IR spectroscopy for handling the quantitative and qualitative problems associated with analyzing food and agricultural products, such as Sudan dye adulteration [24], red wine authenticity assessments [25], and phenolic determination in moringa powder [26]. The examination of chemical information present in food samples by direct observation of spectra is not completely valid and meaningful because of the similarities observed among the peaks; thus, the spectral data are often combined with a chemometric algorithm to extract the hidden chemical information present in scanned samples. Furthermore, some investigations [27,28,29] have already been conducted in the past using FT-IR spectroscopy in conjugation with suitable chemometric algorithms, which clearly demonstrate that FT-IR spectroscopy is a robust technique for quantitative analysis. In addition, in recent years, the development of a hand-held FT-IR spectrometer with improved resolution and better limit of detection was made possible by the advancement in equipment technology in recent years [30]. In order to screen spiked olive oils up to a 10% concentration range, Pan [31] used a hand-held FT-IR spectrometer with chemometrics tools.

The environment for processing highly dimensional data and comprehending complex global problems has fundamentally transformed in recent years as a result of the development of deep learning technologies. A convolutional neural network (CNN), which is usually developed to handle complex spectrum data, is one of the most well-known and widely used deep learning algorithms. A CNN has a number of important advantages over traditional machine learning methods, such as partial least squares regression or other regression techniques such as support vector regression, including the ability to incorporate preprocessing, feature extraction, handling complex datasets, and identification during model training from one end to another without the need for manual adjustment in a single architecture [32]. In the past, CNNs have been considered as a powerful deep learning analysis tool for solving the classification problems associated with image datasets [33,34]. However, the application of a CNN is often limited when dealing with spectral data, which is usually one-dimensional. Since, the FT-IR spectral data are normally provided as a collection of observations made at various wavelengths and their related intensities, these measurements are sequential and have a specific order. The 1D CNNs are designed to process sequential data, and they can effectively capture the local patterns and relationships among the adjacent wavenumbers in the FT-IR spectra. On the other hand, MLPs are better suited for processing tabular data, where each feature represents a distinct variable, and there is no inherent order among them. Therefore, using MLPs to process FT-IR spectral data may not be as effective as using a 1D CNN. Additionally, the one-dimensional CNN model is easy to train, the weights are smaller, it can handle the data present in high dimensional space, and can also go deeper compared to an MLP.

In the past, studies utilized the application of one-dimensional CNNs, such as Raman spectroscopy, to assess the quality of olive oil spiked with vegetable oil [35], perform noninvasive gastric cancer screening based on Raman spectroscopy [36], and find pesticide residues in black tea using fluorescent hyperspectral technology [37]. However, no study has been conducted so far that utilized the combination of a 1D CNN and FT-IR spectroscopy for the quantitative analysis of melamine and cyanuric acid in pet food. Therefore, the main goal of this study was to investigate the feasibility of FT-IR spectroscopy for the nondestructive evaluation of melamine and cyanuric acid in pet food using a combination of machine learning and deep learning approaches. To create the best prediction model, partial least squares regression (PLSR), principal component regression (PCR), and a net analyte signal (NAS)-based methodology, called hybrid linear analysis (HLA/GO), were employed as machine learning techniques, and their effectiveness in terms of both precision and error was compared with that of a one-dimensional convolutional network (1D CNN).

## 2. Materials and Methods

### 2.1. Chemicals

In this study, melamine and cyanuric acid were purchased from Sigma-Aldrich (St. Louis, MO, USA), with 99% purity. Pet food of one variety was obtained from a nearby animal hospital.

### 2.2. Preparation of Fraudulent Pet Food Samples

The pet food samples were obtained from the hospital in solid crystal form, and they were first turned into a powder using a grinder before sample preparation. The powdered samples were dried in oven for 2 h prior to mixing to avoid the interference of moisture present in the samples. Subsequently, different adulterant concentrations were prepared using the dried powdered pet food. In this study, melamine and cyanuric acid were artificially added to pet food at the different concentrations (*w*/*w*) of 0, 0.1,0.5, 0.8, 1, 2, 3, and 4% using an electronic balance (model AR2130, Ohaus Corp., Parasippany, NJ, USA). Samples of each concentration were placed inside snap cap vials that were later transferred onto a high-speed shaker (Vortex-Genie 2, Scientific Industries, Inc., model G560, Bohemia, NY, USA) so that they were uniformly mixed. During the FT-IR spectral measurements, ten replicates of pure and spiked pet food with a total weight of 30 g were chosen.

### 2.3. FT-IR Spectral Data Collection

A Nicolet 6700 FT-IR spectrometer (Thermo Scientific Co., Waltham, MA, USA) was used to obtain the FT-IR spectra of the pet food samples. The attenuated total reflectance sample mode was available on the spectrometer. A deuterated triglycine sulfate detector and potassium bromide beam splitter were the other components of this system, both of which were managed using OMNIC software (version 7.0). A sample was applied to the surface of the diamond crystal sampling plate during spectral acquisition from the 4000 to 400 to cm^−1^ spectral range. After each scan measurement, the diamond crystal was cleaned with ethanol to reduce fluctuations caused during data collection from the previous sample. A background scan was performed using an empty diamond crystal plate on both pure and contaminated pet food samples. A total of 32 scans were collected for each sample at 4 cm^−1^ spectral intervals, and the averaged spectral data were saved in Excel format for further investigation.

### 2.4. Spectral Data Analysis

**Figure 1:**
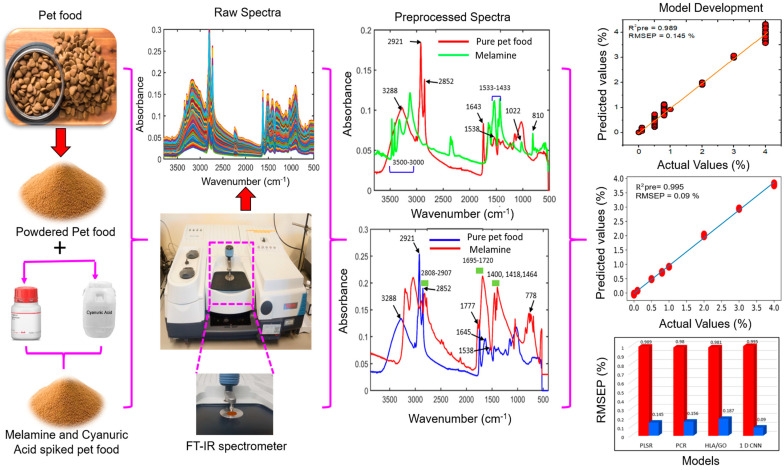
Flowchart of the preprocessing of the FT-IR spectral data of pet food infected with either melamine or cyanuric acid.

When FT-IR spectroscopic data are usually collected, undesirable noise, including experimental drift, particle size, and background effects, can make the data unclean, limit their effectiveness, and negatively impact a model’s forecasting ability. Spectral pretreatment is crucial to avoid noise contamination of the recorded spectral data and to gather precise chemical data. Preprocessing techniques must first be used to rectify the raw spectra. In this study, the different preprocessing steps included range normalization, multiplicative signal correction (MSC), standard normal variate (SNV), and Savitzky–Golay (SG) derivatives (1st and 2nd). The preprocessed data were used to construct the machine learning and deep learning models. The machine learning model was constructed using MATLAB (version 7; Math Works, Natick, MA, USA), whereas the deep learning model was constructed and performed using Python. A flowchart of the spectral analysis process for the pet food samples spiked with melamine and cyanuric acid is detailed in Figure 1.

#### 2.4.1. Prediction Analysis Models Using PLSR, PCR, and HLA/GO

To detect melamine and cyanuric acid in pet food, three widely used multivariate analysis methods, PLSR, PCR, and HLA/GO, were applied in this study, and their performances were compared. PLSR is an effective and quick methodology for process modeling and calibration when the predictor variables are collinear, the measurement data are noisy, the variables are highly dimensional, and the number of samples is fewer than the number of variables [38]. When performing quantitative measurements, PLSR is always considered a robust and strong analytical tool [39,40]. In multivariate analyses, PCR is frequently used to eliminate highly linked predictive variables by combining principal component analysis (PCA) with multivariate linear regression (MLR). First, PCA was used to reduce the number of variables by employing a dimension reduction method. The MLR model was used in the second phase to execute PCR using its optimal number of principal components, as determined by PCA [41]. Furthermore, the quantitative prediction of both chemicals in pet food was also performed using an NAS regression-based HLA/GO algorithms. In [42], a thorough explanation of the mathematical equations is provided. The analyte concentration under examination is precisely proportional to the amount of the signal calculated by the NAS according to the NAS algorithm [43]. As per the method outlined by Goicoechea and Olivieri [42] and Marsili [44], the NAS vector for each sample under examination was established.

#### 2.4.2. Construction of 1D CNN Model Architecture

**Figure 2:**
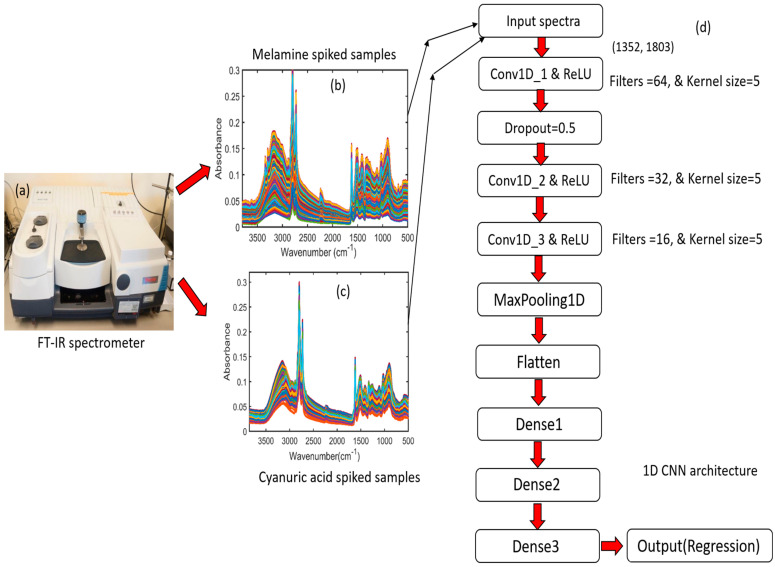
Operational process associated with the 1D CNN model for FT-IR spectral data: (**a**) FT−IR spectrometer; (**b**,**c**) FT-IR spectra for melamine- and cyanuric acid-spiked pet food; (**d**) detailed structure of the 1D CNN model.

One-dimensional data were used as investigation inputs for the 1D CNN model. The features were obtained using a one-dimensional convolution kernel. In this study, to compare the performance of the machine learning methods, such as PLSR, PCR, and HLA/GO, a new deep learning-based quantitative analysis model called 1D CNN was developed for FT-IR spectral data to determine the different concentrations of melamine and cyanuric acid present in pet food. The model consisted of one input layer, three convolutional layers (ConV1D_1, 2, and 3), one max-pooling layer, one flattened layer, three dense layers (Dense 1, 2, and 3), a rectified linear unit (ReLU) as an activation function, and an output layer (regression). The 1D CNN model is illustrated in Figure 2.

Figure 2a depicts the FT-IR spectrometer used to acquire the data, Figure 2b,c display the spectra for pet food spiked with melamine and cyanuric acid, and Figure 2d illustrates the detailed structure of the 1D CNN model. Spectra (Figure 2b,c) were employed individually during the creation of the 1D CNN model for quantitative measurements. The 1D CNN models were implemented using a Jupyter framework and Python 3.11 with TensorFlow. The full specifications of the 1D CNN architecture and all parameters used throughout the training procedure are presented in Table 1 for both chemicals added to the pet food.

For each 1D CNN component, the FT-IR spectra with an input size of 3800–500 cm^−1^ formed the input layer for the 1D CNN model (1352 × 1804). Additionally, three separate 1D CNN layers, each with a different layer of filters of varying sizes, were employed for feature extraction in the 1D CNN model, and to enhance the performance of the created model, alternative kernel sizes and dropout layers were chosen to prevent overfitting. Furthermore, an activation function called the ReLU was implemented, which generally follows a convolutional layer and can be expressed by the following equation:

f(x) = max (0, wx + b)(1)

where x represents the feature maps of the convolutional layer, w is the weight factor, and b is the bias. Finally, the most popular pooling approach is max pooling, which is primarily used to reduce the number of dimensions of the extracted features and network parameters. In this investigation, the 1D CNN network used a max-pooling value of two. In addition, the root mean squared error and loss function values were established to assess the effectiveness of the constructed model.

## 3. Results and Discussions

### 3.1. Spectral Interpretation of Melamine-Adulterated Pet Food

**Figure 3:**
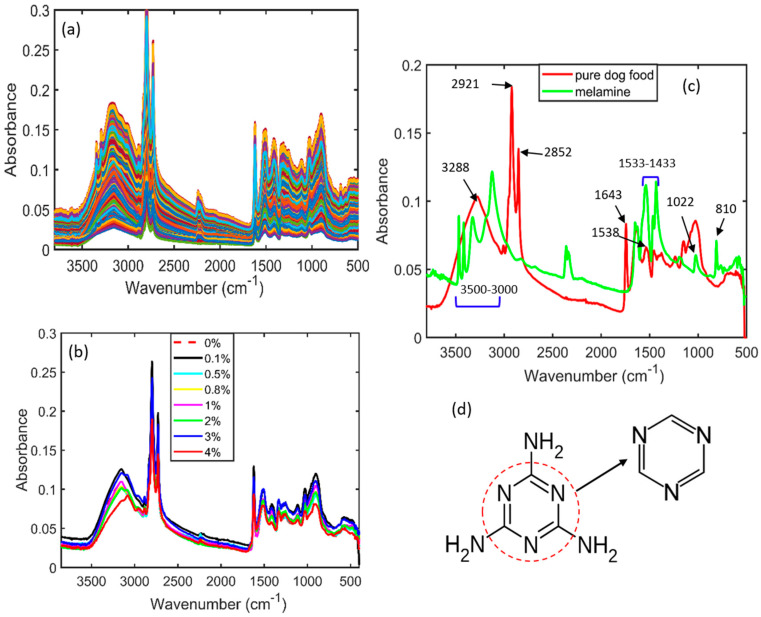
(**a**) Fourier transform infrared raw spectra of adulterated pet food powder samples; (**b**) SNV-preprocessed FT-IR spectra of pet food adulterated with melamine; (**c**) both pure melamine and pure pet food samples; (**d**) chemical structure of melamine and its triazine molecule (indicated with an arrow).

The raw spectra of pet food adulterated with melamine were obtained using an FT-IR spectrophotometer, as displayed in Figure 3a. External factors, such as particle size, background noise, and light interference, normally arise during FT-IR spectral data collection. These factors result in the generation of overlapping peaks that are directly proportional to minimizing the key information necessary to perform prediction or classification tasks. Spectral preprocessing is, therefore, required to produce high-quality data by eliminating background noise and overlapping peaks. Figure 3b shows the SNV-preprocessed spectra of pet food adulterated with melamine at eight different concentrations. The spectra were plotted between wavelengths of 3800 and 500 cm^−1^, because there was no pertinent information below 500 cm^−1^ or above 3800 cm^−1^. The standard normal variate (SNV)-preprocessed spectra for each of the seven melamine concentrations added to pet food are shown in Figure 3b. These spectra did not provide distinct peak information. To determine the main spectral differences between pure melamine and pure pet food, the FT-IR spectra of each were plotted in Figure 3c. The FT-IR spectrum of pure melamine shows a distinct characteristic of absorption peaks at 3000–3500 cm^−1^, 1643 cm^−1^, 1022 cm^−1^, 810 cm^−1^, and 1433–1533 cm^−1^. The stretching vibration of –NH_2_ is linked to the absorption peaks designated at 3000–3500 cm^−1^ (amine group). The triazine ring of melamine, which is shown in Figure 3d with an arrow, is related to the peaks at 1643 cm^−1^, 1022 cm^−1^, and 810 cm^−1^ [45,46]. In addition, the spectral absorption peaks observed at 1433–1533 cm^−1^ are linked with the stretching vibrations of the C-N functional group. However, pet food contains proteins and lipids that also produce infrared absorption peaks. The spectral peaks located at 3288, 1643, and 1538 cm^−1^ represent proteins, whereas the high-intensity peaks observed at 2921 and 2852 cm^−1^ show the sensitive regions of lipids [47]. Since, the objective of this research was to ascertain the presence of melamine and cyanuric acid in pet food, parameters such as proteins and lipids, as well as their effects, were, thus, not given much credence.

### 3.2. Spectral Interpretation of Cyanuric Acid-Adulterated Pet Food

**Figure 4:**
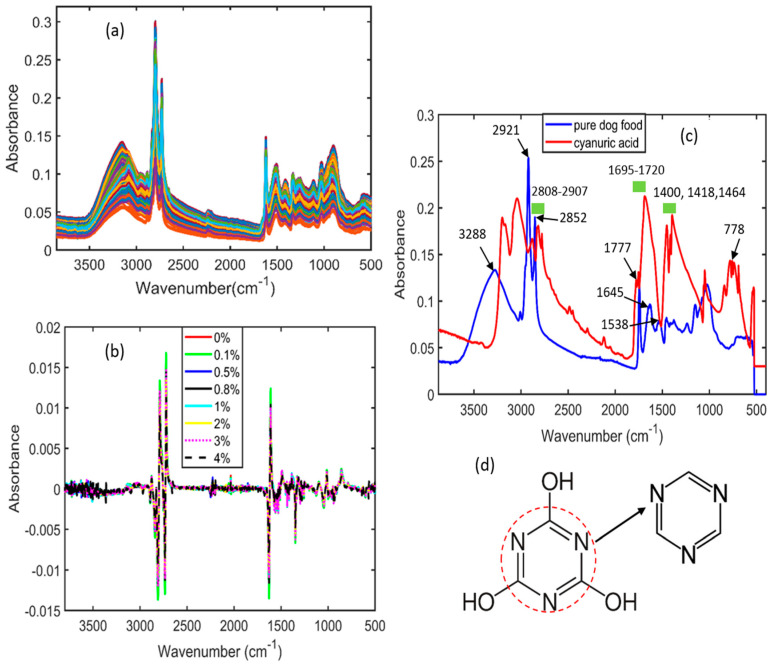
(**a**) Fourier transform infrared raw spectra of adulterated pet food powder samples; (**b**) SG-1-preprocessed FT-IR spectra of pet food adulterated with cyanuric acid; (**c**) both pure cyanuric acid and pure pet food samples; (**d**) chemical structure of cyanuric acid and its triazine molecule (indicated with an arrow).

Figure 4a represents the original (raw) spectra of cyanuric acid-contaminated pet food that were generated using an FT-IR spectrometer. Similar to the melamine-contaminated pet food FT-IR spectra shown in Figure 3a, the raw spectra of cyanuric acid-contaminated food resulted in overlapping peaks and noise (Figure 4a). Figure 4b shows the Savitzky–Golay first derivative (SG-1)-preprocessed data with seven different concentrations, which also could not display clear spectral regions owing to fewer differences observed among the peaks. Therefore, to determine the main spectral differences, the FT-IR spectra of pure cyanuric acid and pure pet food were plotted, as shown in Figure 4c. The spectral regions identified at 3288, 1645, and 1538 cm^−1^ are associated with the normal proteins in pet food, whereas the peaks observed at 2921 and 2852 cm^−1^ indicate their lipids [47]. The FT-IR spectra of pure cyanuric acid is shown in Figure 4c, which exhibited a variety of distinctive peaks over a wide range of wavenumbers. While the peak at 778 cm^−1^ is related to the out-of-plane bending modes, the peaks at 1400, 1418, and 1464 cm^−1^ are associated with the in-plane stretching vibrations of the s-triazine ring, as illustrated in Figure 4d. In addition, pure cyanuric acid showed a number of additional distinct spectral vibrations at 3440, 3210, 2807–2907, 1777, and 1752 cm^−1^ that were associated with the free N-H asymmetric stretching vibration and the H-bonded N-H group between the amine and imine sites, stretching vibrations of the amido group produced from N–H bonds, and carbonyl stretching vibrations [48].

### 3.3. Dirichlet Distribution Algorithm

**Figure 5:**
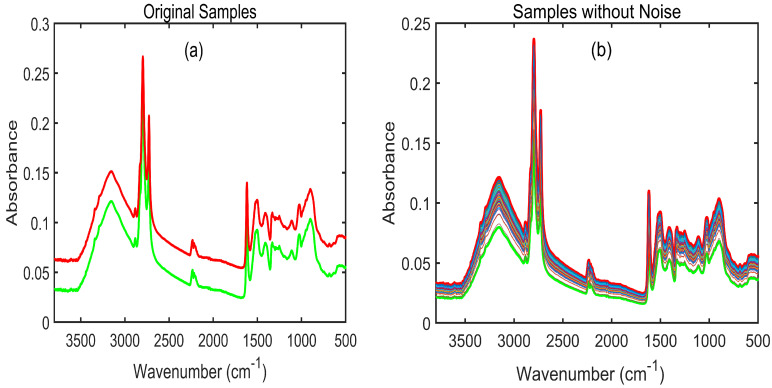
Application of the Dirichlet distribution algorithm on the melamine-contaminated pet food: (**a**) FT-IR spectra created for two replicates (1_1 and 1_5) for one group of samples (0.1%) of the melamine-contaminated samples; (**b**) 1352 mixed-sample FT-IR spectra for one concentration generated using the Dirichlet distribution.

**Figure 6:**
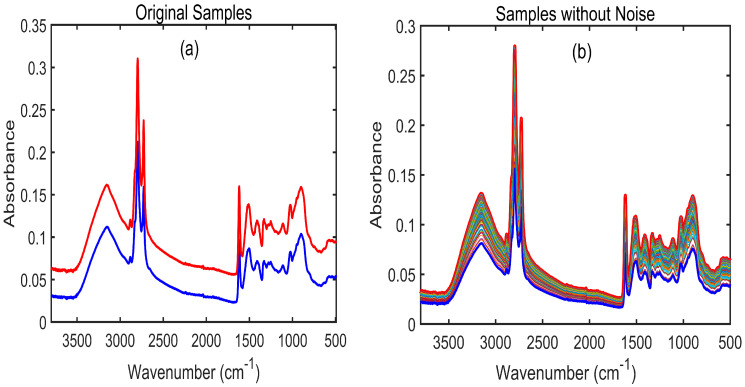
Application of the Dirichlet distribution algorithm to cyanuric acid-contaminated pet food: (**a**) FT-IR spectra created for two replicates (2_1 and 2_6) for one group of samples (1%) of cyanuric acid-contaminated samples; (**b**) 1352 mixed-sample FT-IR spectra for one concentration generated

In this study, 400 samples of FT-IR spectral data were acquired for eight different concentrations of pet food. The number of samples selected during model development played an important role in constructing an efficient model. To avoid the influence of underfitting, 1352 artificial samples were created using the Dirichlet distribution algorithm [49] and utilized for the construction of the machine learning and deep learning models. This algorithm was applied to pet food contaminated with melamine (Figure 5) and pet food contaminated with cyanuric acid (Figure 6). In this context, “original samples” refer to the 1352 spectra created for two replicates during the collection of the FT-IR spectral data (i.e., 1_1 and 1_2 % samples, respectively, in Figure 5a and Figure 6a), while “sample without noise” refers to the 1352 spectra that were preprocessed after the artificial data were generated (Figure 5b and Figure 6b).

### 3.4. Model Development for FT-IR Spectroscopy

The 1352 artificial samples were created with the Dirichlet distribution algorithm, and these samples were then used to create discriminating and prediction analysis models utilizing machine learning models. The data correspond to eight groups of concentrations that were split into calibration and validation groups consisting of 800 samples (100 samples for each concentration) and 552 (69 samples for each concentration), respectively. Next, all three regression models, PLSR, PCR, and Mas-based HLA/GO, were constructed using the calibration set and further tested using the validation set for predicting the presence of melamine or cyanuric acid in pet food samples. Choosing the appropriate number of LVs or factors to take into consideration is an essential step in the calibration model’s production. The quantity of LVs could have a large effect on the model. Incorrect LV selection can result in “overfitting” or “underfitting”, which suppresses spectral information and model interpretation and causes spectral noise in the regression model. Therefore, it is crucial to choose an ideal number of LVs to avoid these weaknesses in the model. In this study, the lowest value of the root mean square (RMS) during the cross-validation (CV) procedure was used to determine the ideal number of LVs.

For the evaluation of any model’s performance, the selection of statistical parameters is crucial. Coefficients of determination, also known as R^2^, evaluate how well the fit accounts for the data’s variation. A good fit is indicated by values close to 1 for the coefficient of determination, which has a range of 0 to 1. However, Fearn [50] suggests that for quantitative research, the R^2^ value alone is not sufficient to determine whether the model is well constructed. To address this issue, root mean square errors of calibration (RMSEC), and prediction (RMSEP), as well as bias, are other essential parameters for evaluating the performance of a model. The absolute fit of the model to the data, or how closely the observed data points match the model’s predicted values, is shown by the RMSE, which is the square root of the variance of the residuals. The primary criterion for assessing a model’s performance in regression analysis is the RMSE, which is a useful indicator of how correctly the model predicts the response. A good model, however, has high R^2^ values and low bias and error values. Equations (2)–(4) were used to calculate the statistical metrics R^2^, RMSE, and bias, respectively:

(2) $$R^{2}=\frac{\sum_{i=1}^{n}{\left(y_{i}-y^{\Lambda }{}_{i}\right)}^{2}}{\sum_{i=1}^{n}{\left(y_{i}-y^{-}{}_{i}\right)}^{2}}$$

(3) $$\mathrm{RMSEP}=\sqrt{\frac{1\;\sum_{i=1}^{n}{\left(y_{i}-y^{\Lambda }{}_{i}\right)}^{2}}{n}}$$

(4) $$\mathrm{Bias}=\frac{1}{n}\sum_{i=1}^{n}\left(y_{i}-y^{\Lambda }{}_{i}\right)$$

### 3.5. PLSR, PCR, and HLA/GO Prediction Results for Melamine in Pet Food

**Figure 7:**
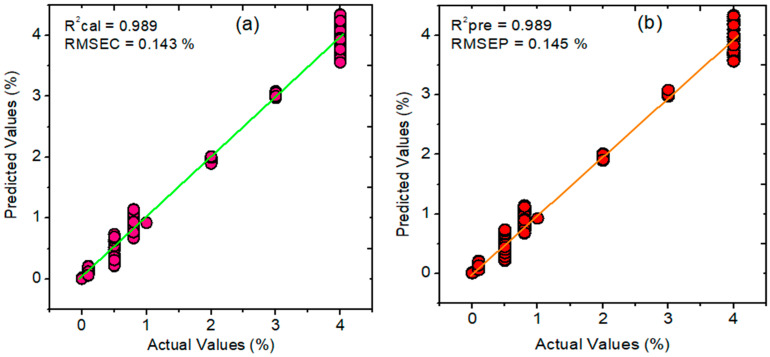
Regression plots developed using PLSR for melamine contaminated pet food: (**a**) calibration; (**b**) prediction plots. RMSEC, root mean square error of calibration; RMSEP, root mean square error of prediction; R^2^, correlation coefficient.

First, a PLSR model was created to perform a prediction analysis of melamine in pet food. This model was created using a variety of preprocessing techniques, including normalization, MSC, SNV, and first and second SG derivatives, with the optimal preprocessing chosen. With the highest correlation coefficient (R^2^ = 0.989) and root mean square error of calibration (RMSEC) of 0.143% for the calibration set, SNV outperformed all other preprocessing techniques, with seven latent variables (LVs). The prediction dataset, on the other hand, achieved a higher correlation coefficient (R^2^ = 0.989) and an RMSEP of 0.145% with a bias of −0.003. Figure 7a,b represents the calibration, and prediction plot constructed using PLSR model.

The PCR and HLA/GO models were created to compare the developed PLSR prediction result. Owing to its superior performance compared with other preprocessing methods, SNV was selected as the best preprocessing method for data pretreatment for both models. However, in the case of the PCR model, the optimum number of LVs was eight, which was chosen based on the minimum error during the calibration and prediction analysis. The calibration dataset’s R^2^ and RMSE were 0.981 and 0.980, respectively, and the prediction dataset’s R^2^ and RMSE were 0.183% and 0.156%, respectively, which were marginally lower than those of the PLSR model.

Contrarily, the HLA/GO model used LVs that were comparable to those used in PCR and produced R^2^ values of 0.978 and 0.981 with RMSE values of 0.183% and 0.156%, respectively. In Table 2, all of the information pertaining to the three models is provided. These results show that the PLSR model accomplished a superior performance to PCR and HLA/GO for detecting melamine in samples of pet food in terms of the R^2^ and RMSE error and with lower bias values.

### 3.6. PLSR, PCR, and HLA/GO Prediction Results for Cyanuric Acid in Pet Food

**Figure 8:**
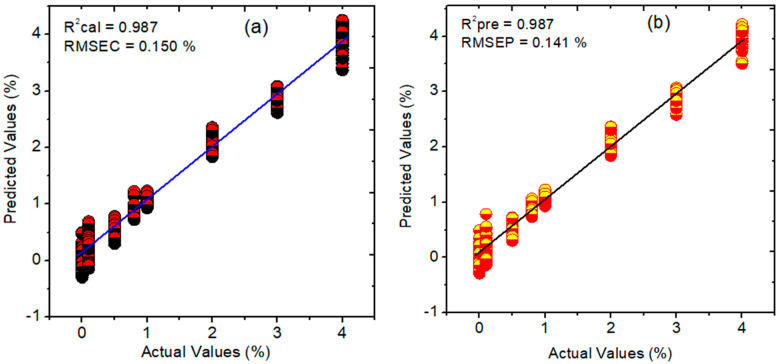
Regression plots constructed using PLSR for cyanuric acid contaminated pet food: (**a**) calibration; (**b**) prediction plots. RMSEC, root mean square error of calibration; RMSEP, root mean square error of prediction; R^2^, correlation coefficient.

The PLSR model was built in conjunction with several preprocessing techniques to assess the presence of cyanuric acid in pet food samples in which the SG-1 outperformed the others and acquired an LV value of 5. The R^2^ and RMSEC for the calibration dataset were 0.987 and 0.150%, respectively, whereas the R^2^ and RMSEP for the prediction dataset were 0.987 and 0.141% with a bias of 0.005 as shown in Figure 8a,b.

To compare the prediction analysis performance of the PLSR, two different regression methods, namely, PCR and HLA/GO, were used for model development. The PCR model results were created using an LV value of eight for the prediction of cyanuric acid in pet food and SNV preprocessing. These findings yielded R^2^ values of 0.955 and 0.956 for the calibration and prediction datasets, respectively. Additionally, for both datasets, the RMSE results calculated using the PCR model were 0.287% and 0.223%, respectively. The HLA/GO model, on the other hand, fared lower than the PLSR and PCR models and produced R^2^ values of 0.945 and 0.951 for both datasets, with higher RMSE values of 0.290% and 0.225%, respectively, when MSC preprocessing was used. The results of all three models are shown in Table 3, which demonstrate that the PLSR model outperformed both of the other models in this investigation at predicting the presence of cyanuric acid in samples of pet food in terms of the R^2^, RMSE, and bias values.

### 3.7. Beta Coefficients for the PLSR Model Developed for Pet Food Contaminated with Melamine or Cyanuric Acid

**Figure 9:**
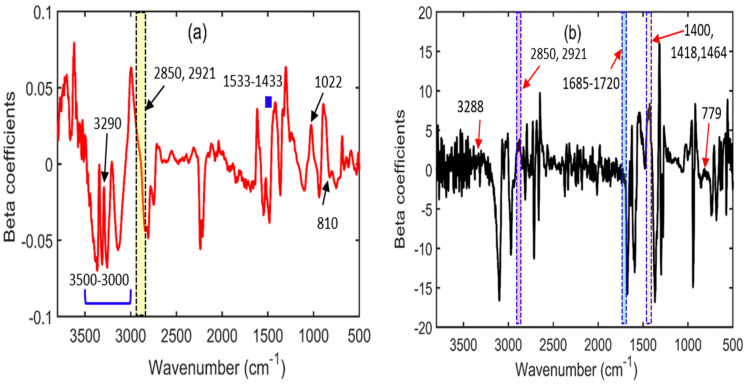
Beta coefficient plots constructed for (**a**) melamine-adulterated and (**b**) cyanuric acid adulterated pet food using the PLSR model for FT-IR spectroscopy.

The PLSR model was used to construct a beta regression coefficient plot for both cases to identify the critical wavenumbers relevant to melamine and cyanuric acid, both of which are present in pet food. The beta coefficient plot produced using the PLSR model for the melamine-contaminated pet food is shown in Figure 9a. Because pet food is rich in proteins and lipids, the beta plots also showed spectral areas for these particular chemicals at 3290, 2850, and 2921 cm^−1^ [47]. The peaks located at 3500–3000, 1533–1433, 1022, and 810 cm^−1^ correspond to melamine vibrations, and the beta plot further offers comparable spectral signatures, as described in Section 3.1. For pet food contaminated with cyanuric acid, the beta plot in Figure 9b shows considerable spectral vibrations, similar to the vibrations described in Section 3.2. Proteins and lipids are connected with the peaks at 3288, 2921, and 2850 cm^−1^ (purple–pink area) [47], while cyanuric acid is related with the peaks at 1685–1720 (blue shaded area), 1400, 1418, 1464 (orange shaded area), and 779 cm^−1^. Therefore, beta coefficient plots are essential for determining the significant wavenumbers of melamine and cyanuric acid.

### 3.8. 1D CNN Analysis for Melamine and Cyanuric Acid Predictions in Pet Food

**Figure 10:**
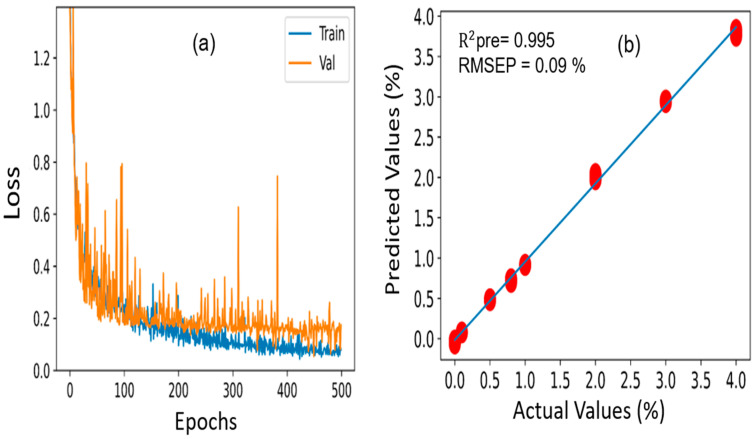
(**a**) Loss function curve and (**b**) prediction analysis results acquired through the 1D CNN model for melamine-contaminated pet food samples.

To compare the prediction performance of the aforementioned machine learning techniques, a new deep learning-based architecture model, known as 1D CNN, was constructed to perform a prediction analysis of pet food samples contaminated with melamine and cyanuric acid. Section 2.4.2 includes a description of how 1D CNN is implemented in detail. Similar datasets of pet food contaminated with melamine and cyanuric acid were utilized in the 1D CNN analysis, and the R^2^ and RMSEP values were used to assess the performance of the model. The 1352 samples were chosen to build the 1D CNN model, which was identical to the machine learning models. Of these, 1148 samples were moved to the calibration set, and the remaining 204 samples were placed in the prediction set. The model is composed of three distinct convolution layers with a 0.5 dropout value. The learning rate for both 1D CNN models (melamine-contaminated and cyanuric acid-contaminated pet food) was 0.001, and the ReLU activation function was selected. During the training process, 500 epochs were selected as the optimum for both pet food samples, and 981,889 training parameters were used. To acquire the greatest prediction performance results, the parameters were carefully adjusted and modified. Figure 10 illustrates the loss function curve and prediction analysis results achieved through the 1D CNN model for the melamine-contaminated pet food. As seen with the loss function curve (Figure 10a), the model began to converge for the training and validation data at 400 epochs. In addition, the developed model acquired an R^2^ value of 0.995 for the prediction dataset, as presented in Figure 10b, demonstrating that the actual and predicted values exhibited strong correlations. The RMSE is a crucial parameter to consider when evaluating the effectiveness of a regression analysis model; however, in our investigation, the RMSEP value achieved using our constructed 1D CNN model was 0.09%, which was lower than our previously developed PLSR and PCR models, highlighting the superiority of the 1D CNN model.

**Figure 11:**
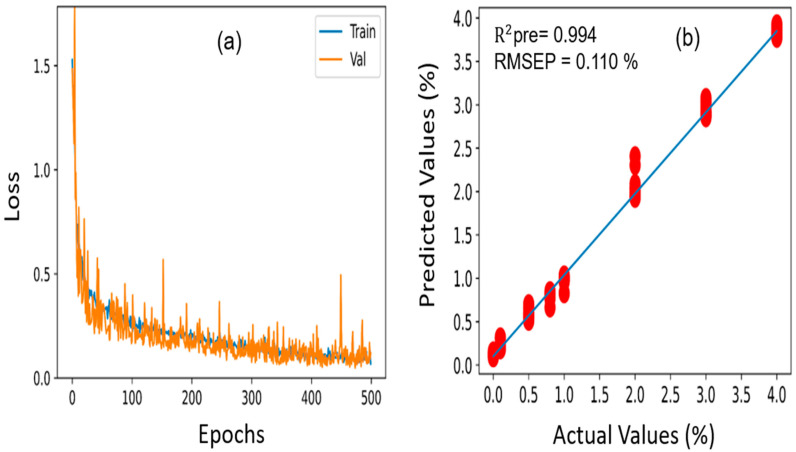
(**a**) Loss function curve and (**b**) prediction analysis results acquired through the 1D CNN model for cyanuric acid-contaminated pet food samples.

On the other hand, Figure 11a, b shows the loss function curve and prediction analysis outcomes for cyanuric acid-contaminated pet food. For the training and validation data, the loss function started to converge after 400 epochs, which is consistent with the findings above. Additionally, the developed model yielded correlation coefficient (R^2^) and RMSEP values of 0.994 and 0.110% for the prediction dataset, demonstrating that the actual and predicted values were in good accordance with one another and were also lower than those of our previously developed PLSR and PCR models.

**Figure 12:**
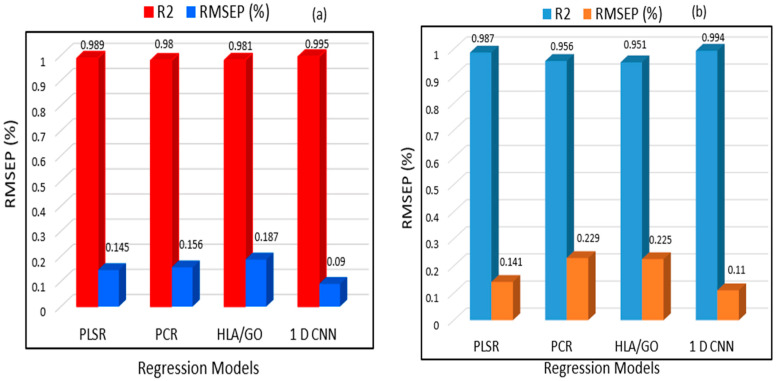
Performance evaluation of the machine learning (PLSR and PCR) and 1D CNN models in terms of the prediction error and correlation coefficient for (**a**) melamine-spiked pet food and (**b**) cyanuric acid-spiked pet food using error bar chart plots.

The two bar chart plots in Figure 12a,b exhibit the performance assessment of the PLSR, PCR, HLA/GO, and 1D CNN models developed for the pet food samples contaminated with cyanuric acid and melamine. Both graphs demonstrate how the RMSEP and correlation coefficient (R^2^) values for the prediction dataset varied. The figures above demonstrate that the 1D CNN model employed in this study completely outperformed the other three regression analysis techniques in terms of R^2^ and RMSEP. The results of the prediction analysis are shown in Table 4 for each of the detailed analysis results for all three regression analysis models on the prediction dataset created in this study.

The prediction analysis findings, as presented in Table 4, clearly support the above statements regarding the 1D CNN model’s performance; hence, it can be suggested that FT-IR spectroscopy, when combined with a one-dimensional convolutional neural network, can be a rapid and reliable analytical tool for the nondestructive examination of melamine and cyanuric acid present in pet food. In earlier findings, Kim [51] used enzyme immunoassay, ultraperformance liquid chromatography, tandem mass spectrometry, and high-performance liquid chromatography with diode array detection to find melamine in pet food with greater limits of detection. In addition, Heller [52] applied zwitterionic hydrophilic interaction chromatography and tandem mass spectrometry for the simultaneous determination and confirmation of melamine and cyanuric acid in animal feed within 0.5 to 50 micrograms/gram (µg/g). However, their use in the real world is frequently time consuming and harmful. This research provides a rapid and simple sample preparation procedure by examining a larger number of samples and developing both machine learning and deep learning models at the same time. Therefore, based on the results, it is clearly confirmed that FT-IR spectroscopy in tandem with a one-dimensional convolutional network (1D CNN) has a high capability for the quick and nondestructive assessment of pet food containing melamine and cyanuric acid.

## 4. Conclusions

In this study, FT-IR spectroscopy was used to perform a nondestructive prediction analysis on pet food contaminated with melamine and cyanuric acid. The models’ development was conducted in the 3800–500 cm^−1^ wavenumber region to reduce spectral irregularity and determine the most significant information related to the additive compounds. The optimal preprocessing method appropriate for this investigation was found by applying various preprocessing techniques to the FT-IR spectrum data that had been affected by both chemicals. The performances of three different machine learning regression models, including PLSR, PCR, and HLA/GO, were compared with that of a 1D CNN deep learning model during the model development process. The prediction analysis results obtained with the 1D CNN model showed R^2^pre values of 0.995 and 0.994 and RMSEP values of 0.090 and 0.110% for the melamine- and cyanuric acid-contaminated pet food samples, respectively. These correlation coefficients and RMSEPs demonstrate the better performance of the deep learning-based model compared to the three machine learning regression models. Based on these findings, it can be stated that FT-IR spectroscopy combined with the 1D CNN deep learning technique offers a strong capability for the quick, accurate, and nondestructive diagnosis of pet food contaminated with harmful chemicals.

Since melamine and cyanuric acid in pet food were found in concentrations of up to 0.1% in this report, more research is required and will be carried out in the future to improve the performance of the deep learning model for the examination of dangerous chemicals in other different types of pet foods that could possibly be present at lower concentrations. Higher-resolution, handheld FT-IR spectrometer applications have grown in popularity in recent years for more effective onsite product quality detection. As a result, further work will be performed to build a portable FT-IR spectrometer with improved resolution and to assess how effective our deep learning model is in detecting numerous adulterants in feed materials. Comparing this study to more traditional destructive methods, such as HPLC, HILIC-UV, ELISA, and zwitterionic hydrophilic interaction chromatography, which provide low detection limits, the developed method appears to hold promise for the efficient, chemical-free, and quick detection of product adulteration, since they do not necessitate a complex laboratory or skilled employees for the analysis.

## Figures and Tables

**Table 1:** 1D CNN architecture details developed for both melamine- and cyanuric acid-adulterated pet food.

Layer (Type)	Output Shape	Parameters	Activation Function
Conv1D_1 (Conv1D) dropout (Dropout) Conv1D_2 (Conv1D) Conv1D_3 (Conv1D) MaxPooling1D flatten Dense_1 Dense_2 Dense_3 Total params: 931,889 Trainable params: 931,889 Non-trainable params: 0	(None, 1799, 64) (None, 1799, 64) (None, 1795, 32) (None, 1791, 16) (None, 895, 16) (None, 14320) (None, 64) (None, 32) (None, 1)	384 0 10,272 2576 0 0 916,544 2080 33	ReLU ReLU ReLU - - - ReLU ReLU -

Conv1D, convolutional layer; Dense, dense layer; ReLU, rectified linear unit.

**Table 2:** Prediction analysis results acquired through the PLSR and PCR models for the presence of melamine in pet food using FT-IR spectroscopy.

Region	Model/Preprocessing	R^2^cal	RMSEC (%)	R^2^pre	RMSEP (%)	LVs	Bias
FT-IR spectroscopy	PLSR/SNV	0.989	0.143	0.989	0.145	7	−0.003
	PCR/SNV	0.981	0.183	0.980	0.156	8	0.012
	HLA/GO/SNV	0.978	0.210	0.981	0.187	8	0.025

FT-IR, Fourier transform infrared; LV, latent variable; MSC, multiplicative signal correction; PCR, principal component regression; PLSR, partial least squares regression; HLA/GO, hybrid linear analysis; RMSEC, root mean square error of calibration; RMSEP, root mean square error of prediction; R^2^cal, calibration dataset correlation coefficient; R^2^pre, predication dataset correlation coefficient; SG, Savitzky–Golay derivative; SNV, standard normal variate.

**Table 3:** Results of the PLSR, PCR, and HLA/GO models’ prediction analysis for the detection of cyanuric acid in pet food using FT-IR spectroscopy.

Region	Model/Preprocessing	R^2^cal	RMSEC (%)	R^2^pre	RMSEP (%)	LVs	Bias
FT-IR spectroscopy	PLSR/SG-1	0.987	0.150	0.987	0.141	5	0.005
	PCR/SNV	0.955	0.287	0.956	0.223	8	0.018
	HLA/GO/MSC	0.945	0.290	0.951	0.225	8	0.030

FT-IR, Fourier transform infrared; LV, latent variable; MSC, multiplicative signal correction; PCR, principal component regression; PLSR, partial least squares regression; HLA/GO, hybrid linear analysis; RMSEC, root mean square error of calibration; RMSEP, root mean square error of prediction; R^2^cal, calibration dataset correlation coefficient; R^2^pre, prediction dataset correlation coefficient; SG, Savitzky–Golay derivative; SNV, standard normal variate.

**Table 4:** Prediction analysis results using all three models for melamine- and cyanuric acid-contaminated pet food samples using FT−IR spectroscopy.

Regression Model	R^2^pre		RMSEP (%)	
	Melamine	Cyanuric Acid	Melamine	Cyanuric Acid
PLSR	0.989	0.987	0.145	0.141
PCR	0.980	0.956	0.156	0.223
HLA/GO	0.981	0.951	0.187	0.225
1D CNN	0.995	0.994	0.090	0.110

R^2^pre, correlation coefficient for prediction; RMSEP, root mean square of prediction; PLSR, partial least squares regression; PCR, principal component regression; HLA/GO, hybrid linear analysis; 1D CNN, one-dimensional convolutional neural network.

## Data Availability

Not applicable.
